# Brain-based symptoms reported in young adults evaluated for cervical spine etiology: a retrospective, cross-sectional clinical study with preliminary data on over 200 patients

**DOI:** 10.3389/fneur.2026.1749266

**Published:** 2026-05-25

**Authors:** Ross A. Hauser, Morgan Griffiths, Ashley Watterson, Danielle Matias, Cam Steilen, Benjamin Ryan Rawlings

**Affiliations:** 1 Caring Medical Florida LLC, Fort Myers, FL, United States; 2 Independent Researcher, Fort Myers, FL, United States; 3 Division of Behavioral and Organizational Sciences, Claremont Graduate University, Claremont, CA, United States

**Keywords:** cerebrospinal fluid, cognitive dysfunction, cross-sectional studies, jugular veins, mental health, spinal instabilities, vagus nerves

## Abstract

**Background:**

“Brain-based” is a term encompassing physical or psychological symptoms that occur due to altered brain function. Brain-based symptoms are on the rise, commonly associated with factors such as electronic device use, chronic stress, and neuroinflammation, but often with an unknown etiology. Here we explore cervical spine abnormalities as a potential underlying factor.

**Objective:**

Based on clinical observations of frequent brain-based complaints, this study aimed to examine whether relatively young adults with brain-based symptoms had patterns of structural cervical spine dysfunction that could explain objective test results and elucidate potential underlying pathophysiologic mechanisms.

**Methods:**

Retrospective, cross-sectional: Clinical data were obtained and analyzed from a chart review that identified 227 consecutive patients who reported at least 1–8 brain-based symptoms (brain fog, headaches, difficulty concentrating, anxiety, depression, panic attacks, rumination, and obsessive thoughts) from January 1, 2022 through June 30, 2022. Reported following STROBE guidelines.

**Results:**

Diagnostic imaging revealed 100% of patients to have radiologically identified forward head posture, 90% with decreased depth of cervical lordotic curve, and 87% to have C1–C2 ligamentous cervical instability (LCI). Significantly decreased vagus nerve and internal jugular vein (IJV) cross-sectional areas (CSAs) were found in 95 and 98%, respectively. Elevated optic nerve sheath diameters (ONSDs) were seen in 99% of patients. Internal jugular vein cross-sectional area at C1 was found to be significantly greater when lying on a cervical orthotic compared to lying supine (*p* < 0.01). Weak but significant relationships were found between C1–C2 ligamentous cervical instability and optic nerve sheath diameter (*r* = 0.14), and C1–C2 LCI and vagus nerve cross-sectional area (*r* = −0.15).

**Conclusion:**

Abnormal cervical spine structure, decreased internal jugular vein and vagus nerve cross-sectional areas, and elevated ONSDs were significantly documented in a cohort of patients reporting brain-based symptoms at an outpatient neck center. The documented cervical structural abnormalities and compression of the carotid sheath structures may represent potential contributing factors or etiological basis of some brain-based symptoms in young adults. Further research is warranted to better understand these associations.

## 1 Introduction

*Brain-based* symptoms are physical or psychological problems that occur due to altered brain function, often having heterogeneous etiologies, meaning different mechanisms may be at play in different individuals (1). Although brain-based symptoms are increasingly reported across various clinical populations, underlying mechanisms often remain unclear or are nonspecific symptoms of modernity, some being associated with social isolation, malnourishment, sedentary lifestyle, sunlight deficiency, sleep deprivation, and internet addiction (2–4). Patients report to our outpatient neck clinic to be evaluated for cervical etiology of their otherwise mysterious or complex symptomatology, typically after seeing many other specialists without receiving a definitive explanation or effective treatment strategy to address their symptoms or conditions. Here we aim to explore potentially under-recognized cervical structural associations or patterns amongst a cohort presenting with various brain-based symptoms.

In this study, we included patients who presented one or more of the following symptoms at initial intake: brain fog, headaches, difficulty concentrating, anxiety, depression, panic attacks, rumination, or obsessive thoughts. Our goal is to explore underlying cervical spine pathology and resultant pathophysiologic mechanisms that could explain some cognitive, neurologic, and mental health symptoms. The purpose of exploring potential underlying mechanisms related to the cervical spine is to present this subset of patients and physicians across multiple disciplines with insight that could provide a foundation for new diagnostic and therapeutic options for otherwise obscure but life-altering brain symptoms.

**Figure 1:**
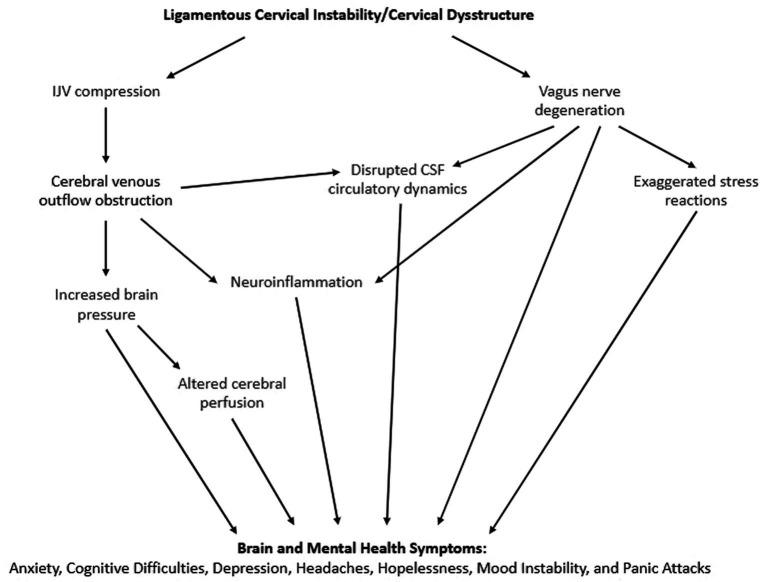
The possible pathophysiology from ligamentous cervical instability causing brain and mental health symptoms.

We hypothesize that cervical spine dysfunction can contribute to abnormal brain physiology primarily by (1) compressing the internal jugular veins, which can reduce total cerebral venous drainage of blood and cerebrospinal fluid (CSF) and lead to increased CSF and intracranial pressure, and (2) causing vagus nerve degeneration or disrupting vagus nerve function, leading to autonomic dysfunction (potentially altered cerebral perfusion, heightened stress sensitivity, mood instability, and neuroinflammation). Both mechanisms potentially result in myriad brain-based symptoms, separately *or* concurrently (Figure 1).

Cervical structural abnormalities were ubiquitous in this patient population, including LCI, forward head posture (FHP), and loss of cervical lordosis, as were IJV compression, vagus nerve degeneration, and elevated optic nerve sheath diameter. The data here support the hypothesis that cervical spine pathology can potentially lead to various brain-based symptoms via several mechanisms.

This preliminary data analysis aims to initiate discussion and encourage further investigation of the proposed mechanisms, rather than draw definitive conclusions. In addition to presenting data on 227 patients, in-office cervical structural analysis protocols and dynamic structural medicine treatment recommendations aimed at improving cervical lordosis and stability are discussed. If some brain-based symptoms have a cervical spine etiology, proper diagnoses could lead to the recommendation for corrective treatment of the cervical spine aimed at relieving neurovascular structures to restore brain health and improve or resolve life-altering symptoms.

### 1.1 Facedown/forward head lifestyle

Given that this cohort excluded previously identified or known causes, including trauma to the cervical spine, yet with cervical pathology prevalent, we speculate a facedown/forward head lifestyle is a contributing factor to the documented cervical spine pathology (5).

The facedown/forward head posture lifestyle of looking down at cell phones or leaning forward into computer screens for multiple hours at a time every day is something that human musculoskeletal systems have only begun to endure in the 21st century, making it likely that we are beginning to see the effects of postural alterations, even in adolescents, now that technology has been prevalent in everyday life for 15–20 years (6). Disorders are even being named depending on the symptoms produced, including “text neck” and “computer vision syndrome” (7, 8). Perhaps many brain-based symptoms are arising in younger-than-expected patients due to poor postural habits and the effects of structural cervical spine changes that have not yet been fully explored.

Smartphone usage is associated with neck pain, and using a smartphone in the *facedown* cervical flexion position doubles the likelihood of experiencing severe symptoms (9). Excessive electronic device (smartphone and computer) use has been strongly associated with an influx of brain-based symptoms (10, 11). In 2025, more than 96% of worldwide internet users report using their mobile phone to connect to the internet (12). The amount of time spent looking at screens each day continues to increase—especially in young people—and with it, socioemotional problems (13, 14). With an increased dependence on electronic devices comes an increase in hours spent in unnatural postures, specifically cervical flexion, putting excessive forces on the cervical spine (especially the posterior ligament complex) and leading to a breakdown of the cervical lordotic curve, a condition known as “cervical dysstructure” (15, 16).

As the neck is the superhighway through which everything that goes into and out of the brain has to traverse, we speculate that significant cervical spine changes can adversely affect optimal brain functioning. These cervical structural changes can cause compression of the carotid sheath, including the internal jugular veins and vagus nerves, and any neurovascular structures running through the neck. Exploring this concept could lead to a reduced number of conditions that lack identifiable causes. Lifestyle was not accounted for in this patient population, however, so this speculation is based on clinical observations with the premise that many patients present without a known cause, trauma, or differentiating diagnosis.

Further research evaluating the breakdown of the cervical spine in relation to brain symptoms should include a comparison group with stable cervical lordotic curves, examine lifestyle risk factors, and investigate the effects of cervical structural correction programs aimed at relieving impaired neurovascular structures to assess if the associated pathophysiology and symptoms improve or resolve.

## 2 Methods

### 2.1 Patients

In this retrospective, cross-sectional study, 227 consecutive patients were identified from a clinical database review of patients reporting to *Caring Medical Florida* from January 1, 2022—June 30, 2022. The patients came to our clinic for specialized assessment of the cervical spine to evaluate potential etiology of otherwise unresolvable conditions. Before identifying qualified patients, the authors first determined 8 brain-based symptoms: brain fog, difficulty concentrating, headaches, anxiety, depression, obsessive thoughts, panic attacks, and rumination. Qualification included (1) aged 20–50, (2) no known cause for symptoms, (3) symptoms lasting for more than 6 months, and (4) reported 1 or more of the 8 symptoms on their initial intake patient questionnaire. Disqualification included (1) having a previously known diagnosis or cause that could explain symptoms, or (2) known preceding trauma to the head or neck.

This study focused on symptoms reported at initial intake and data collected and charted during initial assessment; follow-up data were not included.

All qualified patients had received dynamic structural imaging and “neck vitals” testing following the standard clinical protocol at our outpatient neck clinic, including upright digital motion x-ray (fluoroscopy, DMX) and upright cone beam computed tomography (CBCT) of the cervical spine, pupillometry, tonometry, and ultrasound of the carotid sheath (internal jugular veins and vagus nerves) and the eye (optic nerve). All scanning and testing were done by a radiology technologist or medical ultrasonographer and performed for clinical, not research, purposes. Clinical chart reviews provided all measurements used in this study.

Following DMX and CBCT, measurements were obtained to evaluate for ligamentous cervical instability in the lower cervical spine (C2–C7) in flexion and extension, and the upper cervical spine (C1–C2) in lateral flexion with open mouth views. Measurements of depth of curve (DOC) were obtained to evaluate the state of cervical lordosis, and the C6-atlas interval (C6AI) was used to evaluate structural forward head placement in relation to the lower cervical spine (forward head posture) (Table 1).

**Table 1:** Structural diagnostic imaging and neck vitals analysis methods.

Structural diagnostic imaging methods			
Measurement	Modality and output	Criteria for interpretation	Application
Ligamentous upper cervical (C1–C2) instability, lateral flexion	Upright digital motion x-ray (video fluoroscopy, DMX) of the cervical spine is used for identifying translation of adjacent vertebra (mm)	Normal is <2 mm in any direction	DMX allows structural deviations to be seen *during movements* and in different positions, which can document vertebral translations that could be putting strain on vital structures in the area. The upright position provides a high degree of accuracy which may otherwise be missed.
Ligamentous cervical (C2–C7) instability, flexion and extension			
Depth of curve	Upright cone beam CT of the cervical spine is used to identify distance from the posteroinferior aspect of the C4 vertebra to a line drawn from the posteroinferior aspect of the C6 vertebral body to the peak of the dens of C2 (mm)	Normal is 7–17 mm	Depth of curve (DOC) is used to objectively assess the state of cervical lordosis.
C6-atlas interval (C6AI)	Upright cone beam CT of the cervical spine is used to measure the horizontal distance between the posterior border of the C6 vertebral body and a line drawn perpendicular from the anterior arch of the atlas in the sagittal view (mm)	Normal is <10 mm	C6AI objectively assesses the structural relationship of the atlas in relation to the lower cervical spine (C6) in the sagittal plane, providing an objective measurement for “forward head posture” as it identifies the position of the head, which sits upon the atlas, compared to the lower neck.

Neck vitals analysis methods			
Measurement	Modality and output	Criteria for interpretation	Notes/application
Pupil diameters	NeurOptics NPi^®^-200 pupillometer automatically measures baseline pupil diameter (mm)	Normal is 2–4 mm unilateral (lit environment)	Chronically dilated pupils (mydriasis) can indicate sympathetic nervous system dominance/parasympathetic dysfunction (state of stress on the body).
Pupillary light reflex (percent change)	NeurOptics NPi^®^-200 pupillometer automatically calculates the percent change of the pupil diameter after initiating a flash of light (%)	Normal is 15–30% unilateral	Pupillary light reflex is used in clinical settings to assess dysautonomia. Excessive percent change can be an indicator of sympathetic nervous system dominance.
Internal jugular vein cross-sectional area (IJV CSA)	Canon Aplio a550 ultrasound with 7 MHz linear probe is used to identify cross-sectional areas in multiple positions including seated, supine, and with use of cervical orthotic (mm^2^)	Normal is 90–100 mm^2^ unilateral	Abnormally small IJV CSA is a cause of venous outflow obstruction and indicative of external compression.
Vagus nerve cross-sectional area (vagus nerve CSA)	Normal is >2 mm^2^ unilateral	Atypical vagus nerve CSA is indicative of degeneration and low vagal tone.
Optic nerve sheath diameter (ONSD)	Canon ultrasound, ocular setting is used to identify the optic nerve sheath diameter (mm)	Normal is <6.1 mm unilateral	Elevated optic nerve sheath diameters can be indicative of increased cerebrospinal fluid and elevated intracranial pressure.
Intraocular pressure (IOP)	iCare ic200^®^ tonometer automatically measures intraocular pressure (mmHg)	Normal is <21 mmHg unilateral	Elevated intraocular pressure can be indicative of fluid outflow obstruction and/or elevated venous pressure.

Neck vitals testing provided measurements of pupil diameters to assess for dilation and pupillary light response, optic nerve sheath diameters to evaluate for increased fluid around the optic nerve, internal jugular vein cross-sectional areas to gage structural compression and/or venous outflow obstruction from the brain, and vagus nerve cross-sectional areas to assess for degeneration.

Symptoms at initial intake were reported as frequencies, with the number and percentage of participants experiencing each symptom. The objective test results (quantitative variables) were analyzed as continuous variables. Descriptive statistics included means and standard deviations. No confounding variables were identified or adjusted for in this analysis, which already excluded previous diagnosis and traumatic events, the patient group was relatively young, and it was 48% male, 52% female. This study did not include an analysis of effect modifiers, though modifications such as head position and movement may be considered in future investigations. Patients were selected solely based on symptoms reported and having completed standard imaging and testing at intake, which was performed following standardized protocol, minimizing potential bias.

### 2.2 Statistical analysis

All analyses were conducted in R version 3.1.1 (R Core Team, 2023) using RStudio version 2023.03.1 + 446 (RStudio Team, 2023). Analyses were performed on both the full dataset (*N* = 227) and an outlier-removed dataset (*N* = 200). Statistical significance was defined as *p* < 0.05 for all tests.

#### 2.2.1 Missing data and outlier handling

We did not perform listwise deletion across the entire dataset to preserve sample size and statistical power. Instead, complete case analysis was conducted on a variable-by-variable basis, enabling maximal retention of participants in each analysis. The sample size used in each test is reported accordingly. Univariate outliers were identified using a z-score threshold of ±3 and were excluded from select analyses to reduce the influence of extreme values. Categorical variables were summarized using counts and valid percentages (e.g., number of symptoms), while continuous variables were summarized using means and standard deviations.

#### 2.2.2 Assumption checking and statistical methods

Skewness and kurtosis were evaluated to ensure values fell within acceptable thresholds (±3 and ±10, respectively), and scatter plots were used to confirm linearity between continuous variables. When assumptions for parametric testing were not met, nonparametric alternatives were used.

Pearson correlation coefficients were calculated to examine relationships among variables, including IJV and vagus nerve cross-sectional area, LCI C1–C2 instability, C6AI, and ONSD measures. Wilcoxon signed-rank tests were used to compare IJV cross-sectional areas across 3 positional conditions: C1 supine, C4–C5 supine, and C1 supine on a Denneroll^®^ pillow, due to the skewed distribution of IJV measures.

#### 2.2.3 Confounding, subgroups, and sampling

Given the exploratory and observational nature of the study, no covariates were included, and no formal multivariable adjustment was conducted. Subgroup or interaction effects were not examined. The study did not employ a sampling strategy requiring weighting or design correction, and all analyses were conducted using raw, complete-case data.

#### 2.2.4 Sensitivity analyses

All key analyses were repeated on both the full dataset and the outlier-removed dataset to assess the robustness of results. Where differences between the 2 samples existed, both results were reported and compared for interpretability.

## 3 Results

### 3.1 Participants

A clinical database review of new patients reporting to Caring Medical Florida from January 1, 2022 to June 30, 2022 identified 227 patients to be included in this study (age 20–50, mean 38 ± 8.8 years; 109/227 48% male, 118/227 52% female) (Supplementary Figure 1).

### 3.2 Data

#### 3.2.1 Frequency of symptoms

At initial intake, 205/227 reported brain fog (90%), 202/227 difficulty concentrating (89%), 199/227 headaches (88%), 190/227 anxiety (84%), 167/227 depression (74%), 115/227 obsessive thoughts (51%), 98/227 panic attacks (43%), and 92/227 rumination (41%). Neck pain was reported by 211/227 (93%) of patients (Table 2). Overwhelmingly, 95% of patients who reported at least 1 of the 8 symptoms reported *3 or more* of the 8 symptoms.

**Table 2:** Symptom frequency in cohort of 227 patients with at least 1 of 8 brain-based symptoms.

Symptom	Frequency	Percentage
Neck pain	211	93%
Brain fog	205	90%
Difficulty concentrating	202	89%
Headaches	199	88%
Anxiety	190	84%
Depression/hopelessness	167	74%
Obsessive thoughts	115	51%
Panic attacks	98	43%
Rumination on traumatic events	92	41%

#### 3.2.2 Frequency of abnormal findings

Mean and standard deviation are found in Table 3. All variables were analyzed and reported as totals except age, C6AI, and depth of curve. Structural diagnostic imaging revealed forward head posture (abnormally high C6AI) in 100%, ligamentous cervical instability with lateral flexion in 87%, decreased depth of cervical lordotic curve in 90%, and ligamentous cervical instability in the lower cervical spine (C2–C7) with flexion/extension in 88%. Additional diagnostics, including pupillometry, tonometry, and ultrasound of the carotid sheath and eye revealed excessive pupillary light response (percent light constriction) in 95%, decreased IJV CSA at C1 level and at C4–C5 level in 98% and 78%, respectively, elevated optic nerve sheath diameter in 99%, and small vagus nerve CSA in 95% (Table 4). Much of the data are reported as bilateral totals to streamline results and provide a representative summary, as clinical observation in this patient population indicates that each side is typically affected symmetrically and combining values reduces redundancy without loss of accuracy.

**Table 3:** Mean and standard deviation of test findings in cohort of 227 patients with at least 1 of 8 brain-based symptoms.

Variable (bilateral sums)	*n*	Reference range normal	Mean	Standard deviation (SD)
Age*	227	N/A	37.69	8.79
C6-atlas interval*	226	<10 mm	41.25 mm	13.16
Depth of curve*	224	7–10 mm	2.68 mm	3.53
Internal jugular vein CSA C1 supine total	227	>180 mm^2^	70.81mm^2^	41.75
Internal jugular vein CSA C1 with Denneroll^®^ total	209	>180 mm^2^	106.34 mm^2^	47.91
Internal jugular vein CSA C4–C5 seated total	227	>180 mm^2^	128.42 mm^2^	81.90
Intraocular pressure total	225	<42 mmHg	35.58 mmHg	8.64
LCI C1–C2 lateral flexion total	227	<4 mm	7.23 mm	3.21
LCI C2–C7 extension total**	227	<5 mm	4.41 mm	3.32
LCI C2–C7 flexion total**	226	<5 mm	4.46 mm	3.15
Optic nerve sheath diameter total	227	<12.2 mm	15.38 mm	1.79
Percent light constriction total	227	30–60%	74.39%	9.81
Pupil diameter total	226	<8 mm	10.22 mm	1.58
Vagus nerve CSA total	227	>4.2 mm^2^	2.71 mm^2^	0.81

*All variables are reported as sums except age, C6-atlas interval, and depth of curve. **Total flexion and extension instability at each level from C2 through C7 (instability at C6–C7 was not consistently seen on DMX nor cone beam CT scan). Normally, there is little (<1 mm) anterolisthesis or retrolisthesis with flexion and extension, but what constitutes “excessive” pathological movement is dependent on several variables, including symptomatology. A small number of patients did not have certain test results included in their chart because particular tests are only performed if they are clinically indicated. To avoid inaccurate or misleading data, some clinical measurements were not charted if imaging was not clear enough to draw accurate conclusions.

**Table 4:** Summary of significant objective testing and cervical structural parameters that were abnormal in cohort of 227 patients with brain-based symptoms.

Diagnostic test (bilateral sums except C6AI and depth of curve)	Cut-off normal criteria	Abnormal [%, (#/n)]
C6-atlas interval (forward head)*	<10 mm	100% (226/226)
Optic nerve sheath diameter total	<12.2 mm	99% (225/227)
Vagus nerve CSA total	>4.2 mm^2^	95% (215/227)
Internal jugular vein CSA C1 supine total	>180 mm^2^	98% (222/227)
Percent light constriction total	<60%	95% (215/227)
Pupil diameter total	<8 mm	91% (206/226)
Depth of curve*	7–10 mm	90% (201/224)
LCI Flexion/Extension**	<5 mm	80% (182/227)
LCI C1–C2 total	<4 mm	87% (198/227)
Internal jugular vein CSA C4–C5 Supine total	>180 mm^2^	78% (176/227)
Intraocular pressure total	<42 mmHg	19% (42/225)

* All variables are reported as sums except age, C6-atlas interval, and depth of curve. **Total flexion and extension instability at each level from C2 through C7 (instability at C6-C7 was not consistently seen on DMX nor cone beam CT scan). Normally, there is little (<1 mm) anterolisthesis or retrolisthesis with flexion and extension, but what constitutes “excessive” pathological movement is dependent on several variables, including symptomatology (124). A small number of patients did not have certain test results included in their chart because particular tests are only performed if they are clinically indicated. To avoid inaccurate or misleading data, some clinical measurements were not charted if imaging was not clear enough to draw accurate conclusions.

### 3.3 Additional analyses

#### 3.3.1 Correlations

- Ligamentous Cervical Instability C1–C2 and Optic Nerve Sheath Diameter

**Figure 2:**
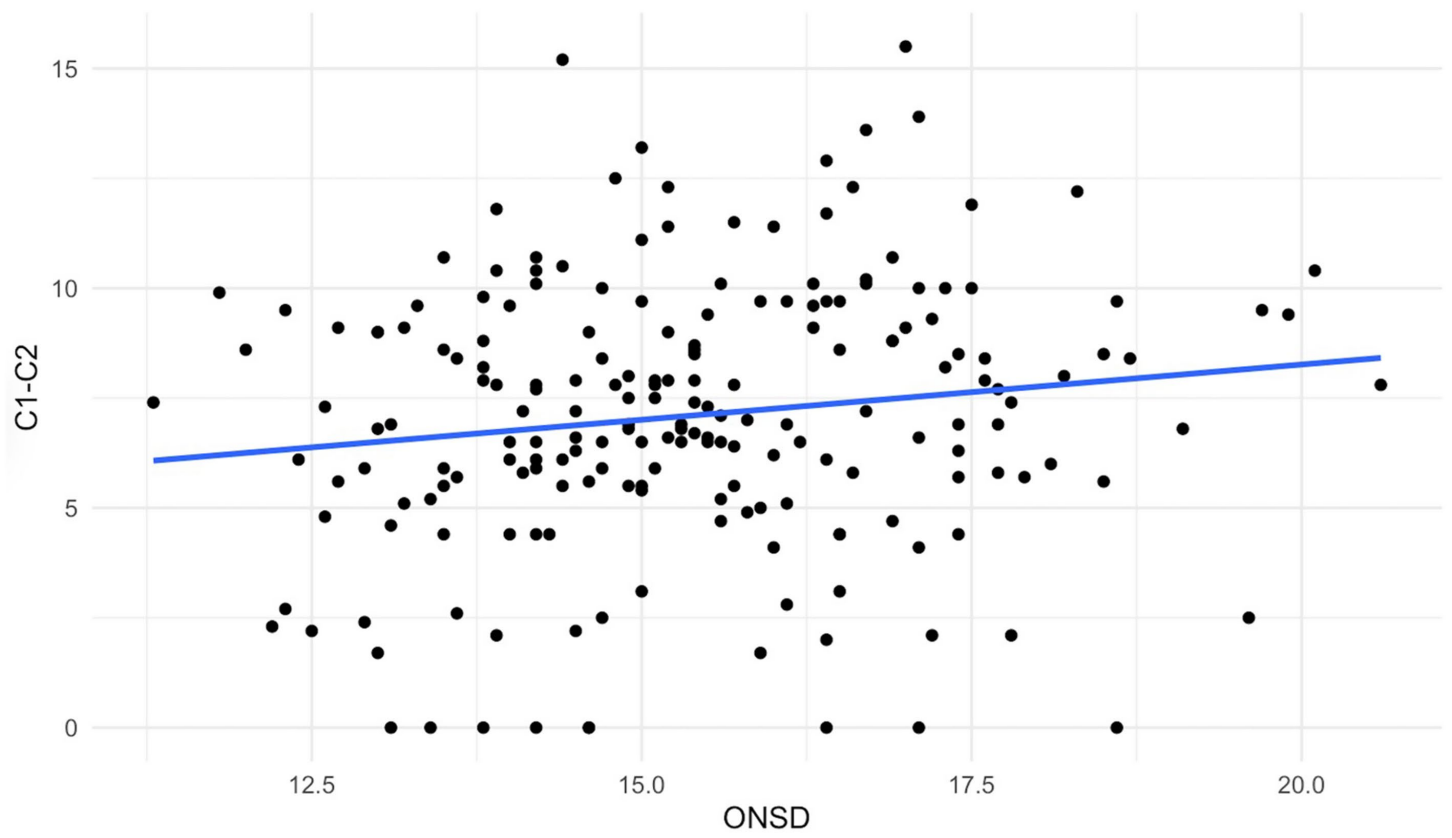
Scatter plot of C1-C2 vs. ONSD.

A Pearson correlation analysis showed a weak but significant positive relationship between LCI C1–C2 and ONSD (−0.14) indicating that *as LCI C1–C2 increases, optic nerve sheath diameter increases* (*R* = 0.14, *p* < 0.05) (Figure 2)

Ligamentous Cervical Instability C1–C2 and Vagus Nerve Cross-sectional Area

**Figure 3:**
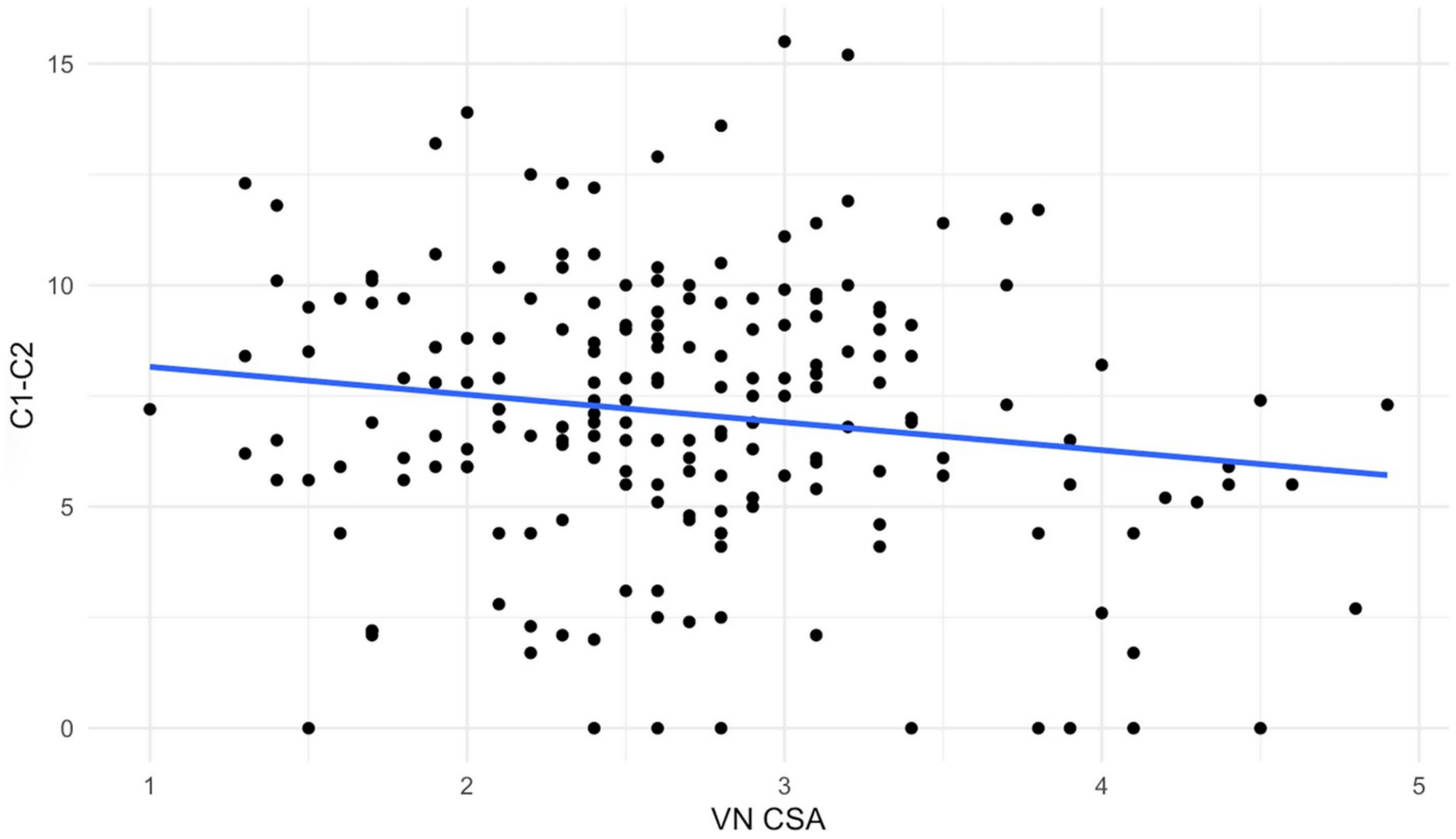
Scatter plot of C1-C2 vs. VN CSA.

A Pearson correlation analysis showed a weak but significant negative relationship between LCI C1–C2 and vagus nerve CSA (*R* = −0.15, *p* < 0.05) indicating that *as LCI C1–C2 increases, vagus nerve CSA decreases* (Figure 3).

#### 3.3.2 Comparisons

**Figure 4:**
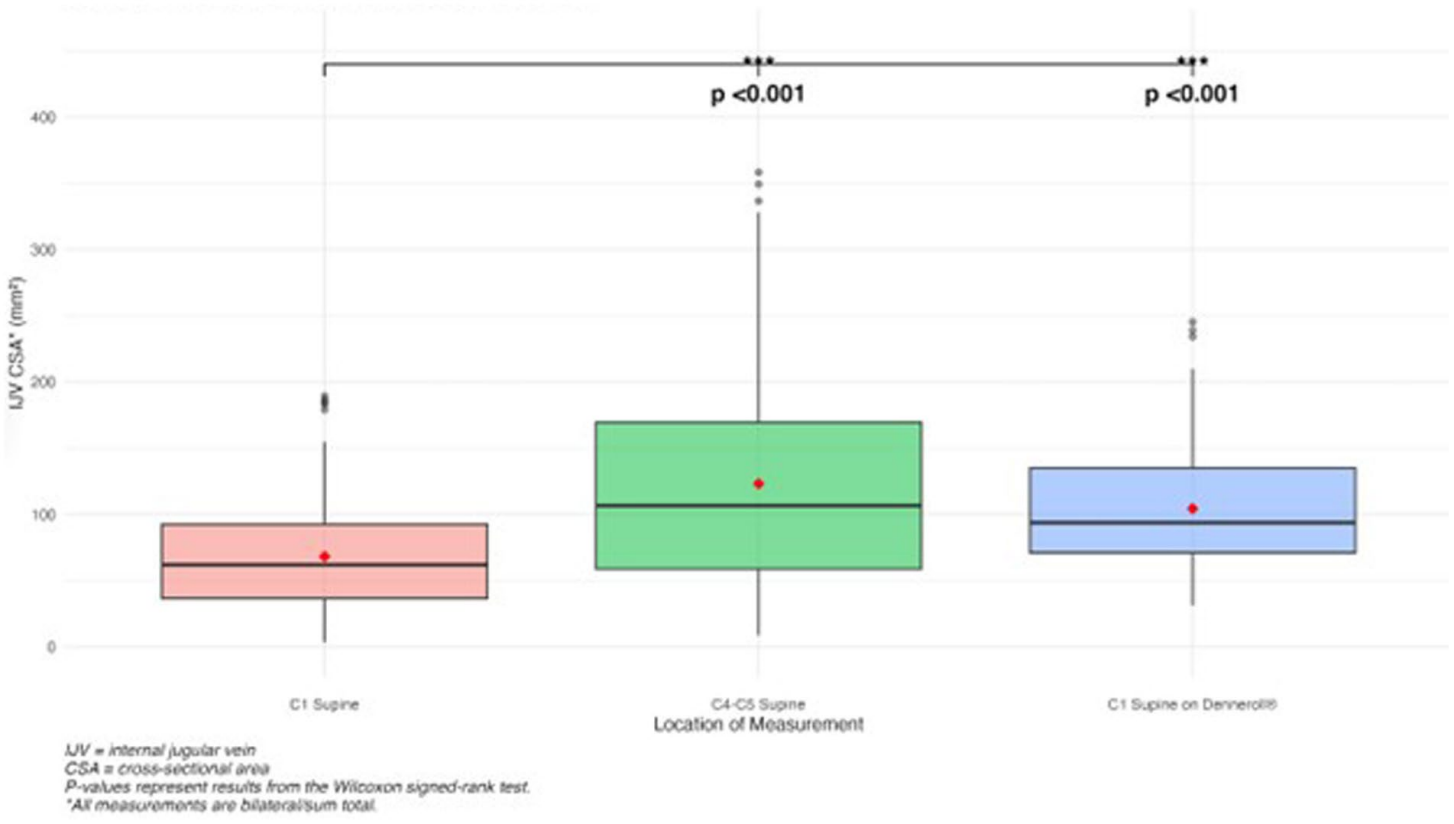
Comparison of IJV CSA by cervical level and device.

- An independent-samples *t*-test was conducted to compare total vagus nerve CSA between our patient sample and a published reference population of healthy controls (17). The mean vagus nerve CSA in our sample (*M* = 2.68 mm^2^, SD = 0.75, *n* = 200) was significantly lower than the value reported for the reference population [*M* = 4.00 mm^2^, SD = 0.78, *n* = 43, *t*(241) = −10.40, *p* < 0.001]. The magnitude of this difference was large, Cohen’s *d* = −1.75, indicating substantially reduced vagus nerve CSA in our clinical sample compared to (healthy) published controls (Table 5).
- A paired *t*-test and a Wilcoxon signed-rank test were performed to compare IJV CSA at C1 supine to IJV at C4–C5 supine. Both the paired t-test and Wilcoxon signed-rank test confirm a significant difference between IJV CSA C1 supine and IJV CSA C4–C5 supine, *t*(199) = −13.67, *p* < 0.001. The mean difference of −55.55 units with a 95% confidence interval (−63.56 to −47.54) indicates that *IJV C1 supine is notably lower than IJV C4–C5 supine*, with consistent findings across both tests, regardless of whether means or medians are compared (Figure 4).
- A paired *t*-test and a Wilcoxon signed-rank test were performed to compare IJV CSA at C1 supine to IJV CSA at C1 while lying on a cervical orthotic, the Denneroll^®^. The results revealed a statistically significant difference between IJV CSA at C1 supine and IJV CSA at C1 with the Denneroll^®^ device, *t*(181) = −10.582, *p* < 0.001. The mean difference was −37.70 mm, with a 95% confidence interval of −38.80 to −26.60. These results suggest that, on average, IJV CSA at C1 while lying on the Denneroll^®^ is significantly higher than IJV CSA at C1 supine (Figure 4).

**Table 5:** Vagus nerve cross-sectional areas in cohort of 84 patients.

Variable	Current study (*n* = 84)	Reference population*(*n* = 43)
Vagus nerve CSA total	2.68 mm^2^	4.00 mm^2^
Mean age	38	42

*Reference population was obtained from Bedewi et al. (17).

## 4 Discussion

### 4.1 Key results

The purpose of this study was to examine cervical spine structure and additional objective test findings in a group of relatively young adults aged 20–50 (52% F, 48% M) reporting brain-based symptoms to evaluate for potential structural etiologies and pathophysiological patterns. We identified 227 patients reporting brain-based symptoms as one of their concerns during their initial intake at our outpatient neck clinic over the course of 6 months. We found significant cervical spine pathology to be overwhelmingly present in this cohort of patients, accompanied by significantly atypical objective test results, that could elucidate an underlying contributing factor or pathophysiology of some brain-based symptoms.

Key cervical structural findings of forward head posture measured by C6AI, loss of cervical lordosis measured by depth of curve, and ligamentous cervical instability in the upper (C1–C2) and lower (C2–C7) cervical spine lead us to believe cervical dysstructure—breakdown of the cervical curve—is the cause of the prevalently decreased internal jugular vein CSA at C1–C2 (indicating bony compression) and small vagus nerve diameters, suggesting degeneration or atrophy. The IJV compression could potentially explain the elevated ONSD—a known indicator of increased intracranial pressure—seen across the entire cohort, and possibly some cases of increased intraocular pressure (18–20). Vagus nerve degeneration could potentially explain autonomic imbalances (parasympathetic dysfunction) that are commonly associated with conditions which include brain-based symptoms (21, 22).

Overall, we find cervical dysstructure (LCI, forward head posture, and loss of lordosis) in our clinic to be strongly associated with IJV compression and vagus nerve degeneration, which could be partial contributors or primary mechanisms of various brain-based symptoms, due to the numerous possible outcomes of cerebral venous obstruction and dysregulation of the autonomic nervous system. These preliminary findings suggest potential relationships worth further investigation, especially in the clinical context of otherwise elusive symptoms and complex cases. *As this is a preliminary investigation, we did not aim to assign distinct mechanisms to individual symptoms, we only strived to observe frequencies or patterns associated with the entire cohort.*

### 4.2 Lifestyle can cause LCI, forward head posture, and loss of lordosis

The average American spends more than 8 hours a day using digital devices (23). The facedown/forward head posture while using digital devices strains the cervical spine, which eventually leads to weakened ligaments and changes in the cervical structure that can impinge on vital neurovascular structures running through the neck (24). Biomechanically, a stable cervical lordotic curve can resist large forces, as 36% of the compressive load is absorbed by the anterior column and 64% by the posterior facets; but as the cervical curvature breaks down, the forces reverse, and a force distribution dysfunction occurs, creating a scenario for ligamentous injury, particularly in the upper cervical region (25, 26). It is permissible to assume that the modern lifestyle can instigate LCI, cervical dysstructure, and potentially associated brain symptoms, as mobile phone usage is associated with degenerative neck conditions, and forward head posture is associated with increased stress-related brain activity (excessive gamma wave activity) (27, 28).

When the cervical spine is in flexion, the entire posterior ligament complex of the cervical spine is being stretched. The modern lifestyle dependent on cell phone and computer use leads to a process known as “creep,” where the posterior ligament complex—especially the capsular ligaments—becomes overstretched and eventually weakened. In this patient population, the prevalent forward head posture (decreased C6AI) and decreased cervical lordotic curve (decreased DOC) are likely due to hours spent in the facedown/forward head posture.

**Figure 5:**
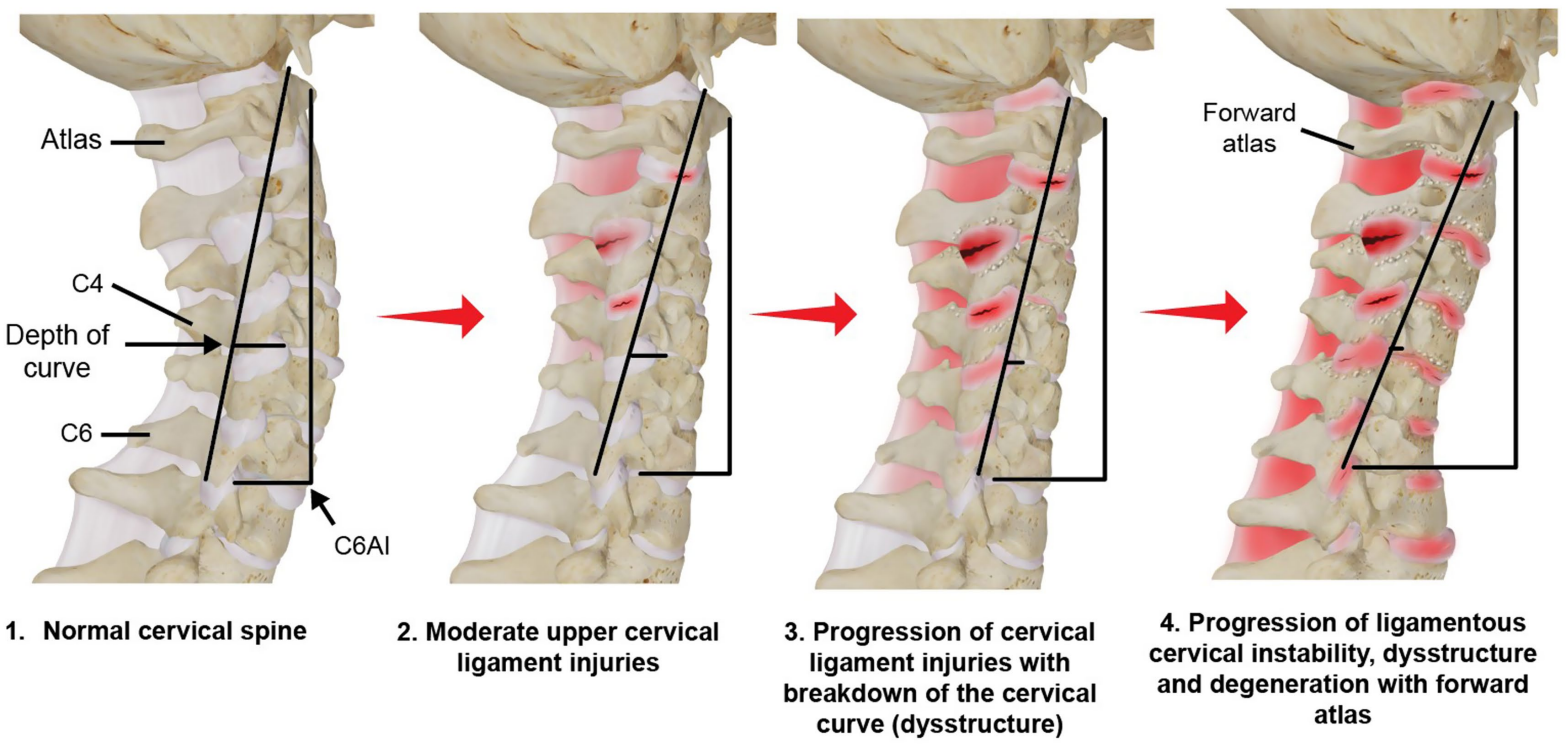
A measurement of the C6-atlas interval (C6AI) documents the “neck component” of forward head posture by measuring how forward the atlas is in the sagittal plane compared to the lower cervical vertebrae. Optimal is <10 mm.

Cervical dysstructure describes the effects of the degenerative process on the cervical spine due to ongoing unnatural forces and resultant ligamentous cervical instability: a breakdown of the cervical curve, a forward shift of the atlas in comparison to the lower cervical spine, and eventual straightening of the spine and kyphosis (5) (Figure 5). Cervical dysstructure was universally present in this patient cohort, with 100% exhibiting a forward shift of the atlas in the sagittal plane (increased C6AI), and 90% presenting with loss of cervical lordosis (decreased DOC).

We objectively identified forward head and neck posture by measuring the C6AI: the horizontal distance between the posterior border of the C6 vertebral body and a line drawn perpendicular from the anterior arch of the atlas in the sagittal view. C6AI assesses the alignment of the atlas in relation to the lower cervical spine (C6).

Forward head posture is often identified by the placement of the head in relation to the shoulders, but clinical radiographic measurements allow for objective documentation of sagittal cervical alignment, comparing the anatomy in the upper cervical spine (C1, atlas) to the lower cervical spine (C6) (29). Several studies have evaluated forward head posture using C2–C7 cervical sagittal alignment, craniocervical angle, or the C2–C7 Cobb angle (which also analyzes cervical lordosis), but we use the C6-atlas interval with a cutoff normal value of 10 mm to determine the forward shift of the atlas in comparison to the lower cervical spine, given that based on clinical observations, neurovascular structures are most often affected at the level of the atlas when the head and cervical structure shift forward (1, 30). Another effect of loss of the normal cervical lordotic curve is that the physical structures in the neck, including those in the carotid sheath and even in the spinal cord, are stretched. The spinal cord, for instance, elongates by around 1 cm when the cervical spine goes from maximum extension to maximum flexion (31, 32).

DOC is used to objectively identify the depth of the cervical lordotic curve. DOC was measured as the distance from the posteroinferior aspect of the C4 vertebra to a line drawn from the posteroinferior aspect of the C6 vertebral body to the peak of the dens of C2. Normal depth of cervical lordotic curve ranges from 7 to 17 mm, as evaluated by the Borden method (33). It is well documented that the excessive use of technological devices with a forward head posture leads to the loss of cervical lordosis (15, 34).Loss of cervical lordosis not only elongates the cervical spine, but also causes stretch and lengthening of the soft tissues, spinal cord, nerves, and blood vessels that travel through the neck, affecting tension patterns, blood flow, and nerve conduction (35). LCI and cervical dysstructure can be progressive conditions unless the forces of the neck change and cervical structural integrity is restored.

### 4.3 Ligamentous upper cervical instability

LCI was objectively documented in the upper cervical spine in this patient population as the amount of total bilateral overhang of C1 on C2 (in millimeters) during lateral flexion and the lower cervical spine (C2–C7) during flexion and extension. Each patient underwent an upright (standing) digital motion x-ray of the cervical spine, during which each motion was done 3 times to visualize the maximum amount of vertebral overhang (36).

While there are various recommendations as to what denotes cervical spine stability, especially in the craniocervical junction, the overall diagnostic criteria for LCI remain controversial (37–39). LCI is characterized by vertebral translation >2 mm in one direction, defined by the degree of overhang by an adjacent vertebra, though some consider as little as 1 mm to be significant (40, 41). Upright scanning techniques, including MRI, are a more sensitive indicator of soft tissue injury in the upper cervical region (especially with flexion/extension views) than when a recumbent scan is performed (42–45). DMX (fluoroscopy) has potential advantages of real-time kinematics that static imaging techniques such as flexion/extension x-rays or MRIs do not, though there is a lack of standardized thresholds and results can be operator- and technique-dependent with variable reproducibility; as such, it is often viewed as adjunctive or investigational (46, 47).

During FHP, the lower cervical spine is placed in flexion, and to maintain gaze stability, the upper cervical spine hyperextends, making ligamentous C1–C2 facet joint instability more likely (48). The articular facets of the upper cervical spine are basically horizontal in orientation, as they are made for quick motions when the atlas rotates around the dens of the axis; because of this orientation, they have very little inherent stability, and thus rely on the capsuloligamentous attachments at C0–C2 for stability (49). The capsular ligaments of the facet joints of the cervical spine are meant to inhibit flexion, extension, axial rotation, and lateral flexion, which is why we focus on facet joint instability when using motion diagnostic scanning to evaluate for excess movement through the cervical spine (16, 50).

**Figure 6:**
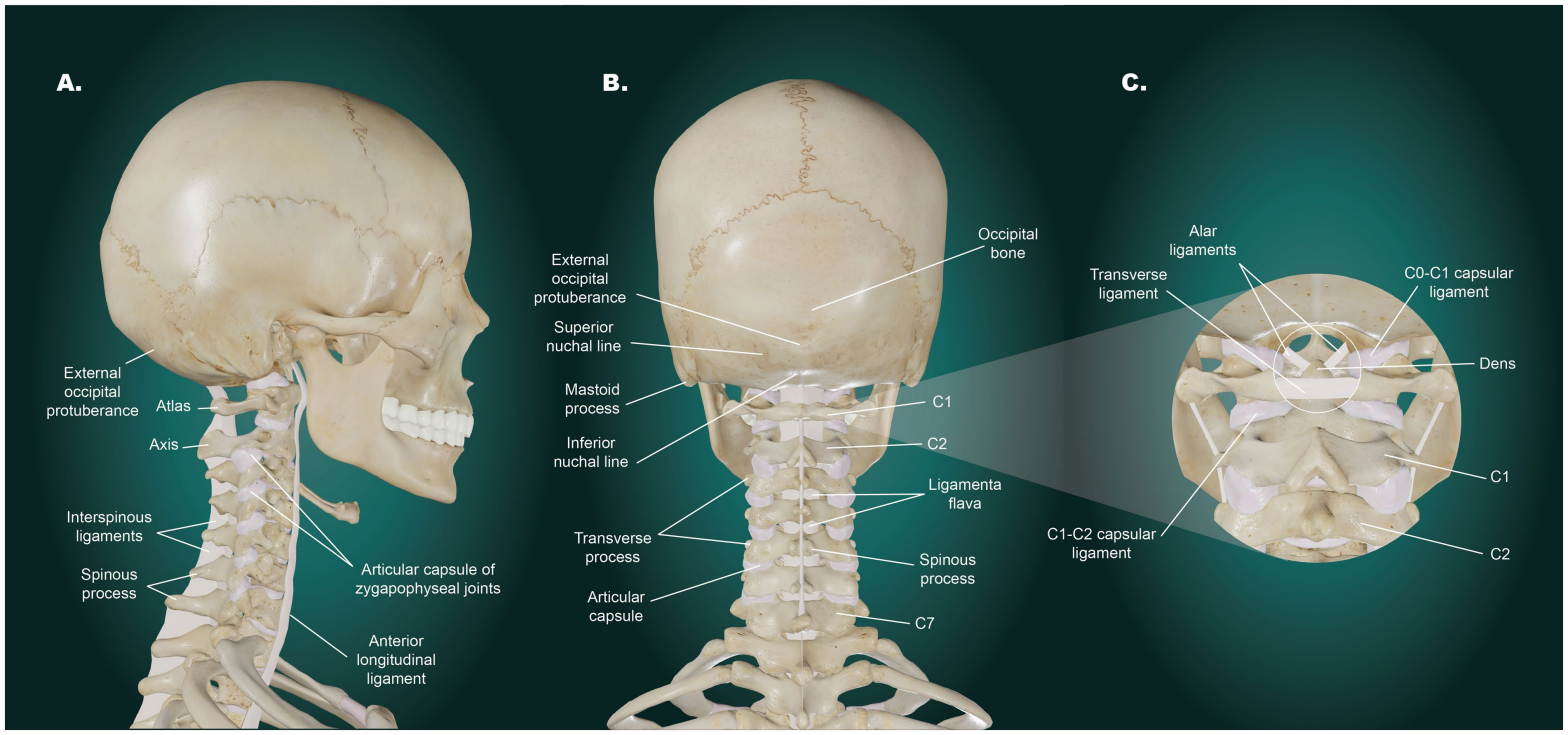
Ligament anatomy of the neck. **(A)** Lateral view. **(B)** Posterior view. **(C)** Upper cervical view.

Ligaments are connective tissues that hold bones together like fasteners. Capsular ligaments are the primary ligamentous supports of the lateral facet joints of C1–C2, distinctly different from the median atlantodens (pivot) joint of C1–C2, which is held together by the transverse and alar ligaments. Ligaments can lose integrity by becoming overstretched due to abnormal use or excessive forces. Should the capsular ligaments lose integrity, the C1–C2 joint is prone to excess movement in lateral flexion and the rotational plane. Compounded by the more stable segments above and below, as well as the weight of the head (10–12 pounds) sitting upon the 2-ounce atlas, C1–C2 is highly susceptible to being the most unstable joint in the cervical spine (Figure 6).

In response to weakened ligaments, the muscles become activated by the ligamento-muscular reflex, which can cause neck pain due to tension or tenderness in the suboccipital and posterior neck muscles: a hallmark complaint in ligamentous upper cervical instability (51). Most patients (93%) in this cohort reported neck pain. Cervical anatomy relating to LCI is further discussed in a previous publication, *The ligamentous cervical instability etiology of human disease from the forward head-facedown lifestyle: emphasis on obstruction of fluid flow into and out of the brain* (5).

Common symptoms associated with ligamentous cervical instability include cracking, clicking, and grinding in the neck with movement, as well as muscle tension, weakness, and headaches (52). Since cervical instability can disrupt nerve signaling and fluid flow between the brain and the body, however, many other disabling symptoms may result, but commonly vertigo, dizziness, tinnitus, migraine headaches, and brain fog (5, 47).

### 4.4 Identifying IJV compression using B-mode ultrasound

**Figure 7:**
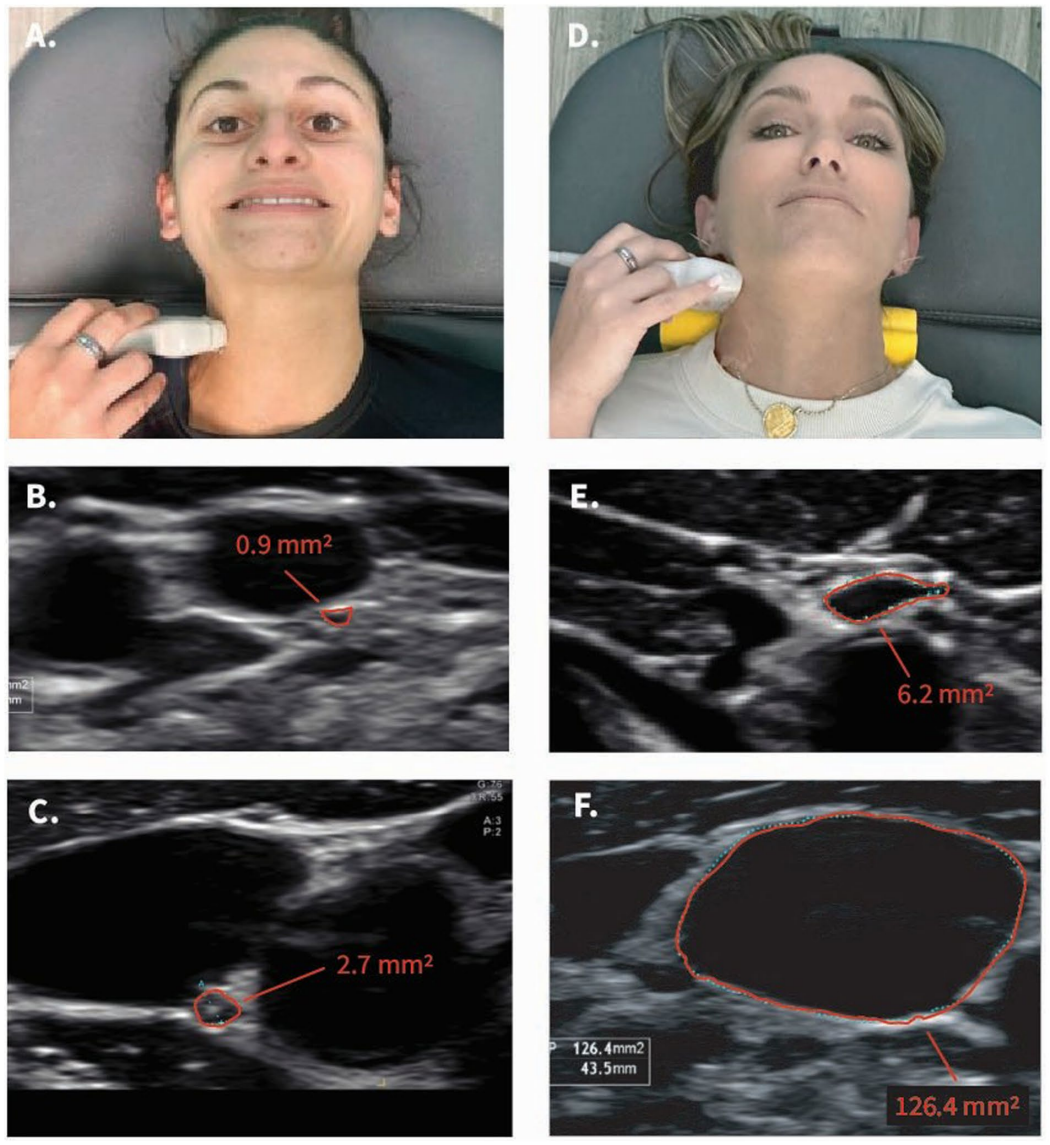
Ultrasound measurement of internal jugular vein (IJV) and vagus nerve as part of full diagnostic workup. **(A)** Probe placement of vagus nerve at C4-C5. **(B)** Abnormal vagus nerve at C4-C5, <1 mm. **(C)** Normal vagus nerve at C4-C5, >2 mm. **(D)** Probe placement of IJV at the C1 level lying on a Denneroll^®^. **(E)** Abnormal IJV, <40 mm. **(F)** Normal IJV, >90 mm.

The internal jugular veins are easily measured using B-mode ultrasound, as utilized in this study (Figure 7). Normal IJV CSA in the supine position is >90–100 mm^2^ and approximately 25 mm^2^ in the upright position (6, 53, 54). Measuring the cross-sectional area of the IJVs in the mid-neck (C4–C5) and upper neck (C1–C2) in both a seated and supine position and with different head and neck positions while using ultrasound can determine if the IJVs are not only being compressed, but by how much and which positions cause the compression.

A comparison between IJV CSA at C1 and C4–C5 supine showed IJV CSA at C1 to be significantly lower in this group of patients with widespread brain-based symptoms. In our cohort, the statistically significant decrease in CSA of the IJVs at the atlas compared to the mid-cervical region (C4–C5 level) suggests that IJV compression can be missed if clinicians only look at the mid-neck region. We suspect the decreased IJV CSA at the atlas is from a breakdown of the cervical curve (measured as DOC, loss of lordosis), forward head posture (measured as C6AI), and ligamentous cervical instability.

The consequence of LCI and cervical dysstructure is often a forward shift of the atlas in 3-D space, putting stretch and compression on the soft tissues and neurovascular contents of the neck, such as the carotid sheath, including the IJVs. We therefore propose that restoration of the cervical curve be the first line of care in treatment of patients’ IJV compression at the atlas when there is documented cervical dysstructure.

Our hypothesis that IJV compression can be treated by correction of the cervical lordotic curve is supported by demonstrating significant improvement in IJV CSA at C1 when lying on a cervical orthotic device, the Denneroll^®^, compared to without (160.3 vs. 70.8 mm^2^, *p* < 0.001). By lying on the Denneroll^®^, the cervical curve is encouraged to go into lordosis. The increased IJV CSAs when lying on the cervical orthotic device (Figure 7) indicate that by following a curve correction program and restoring structural integrity of the cervical spine to maintain proper lordosis, the IJV compression by the atlas should be relieved, potentially accompanied by a reduction in symptoms.

#### 4.4.1 Bony compression of IJV

In this patient population, IJV compression was present at C1 in 98%. IJV compression by elongated styloid bones, a condition known as Eagle’s syndrome, is well established (55). While the current standard of care for symptomatic Eagle’s syndrome is styloidectomy, there is growing interest in IJV compression at the lateral mass of the atlas and whether or not that should also be removed (56). Other documented bony causes of IJV compression are the hyoid bone and cervical osteophytes (57, 58).

#### 4.4.2 IJV compression, cerebral venous outflow insufficiency, and intracranial pressure

**Figure 8:**
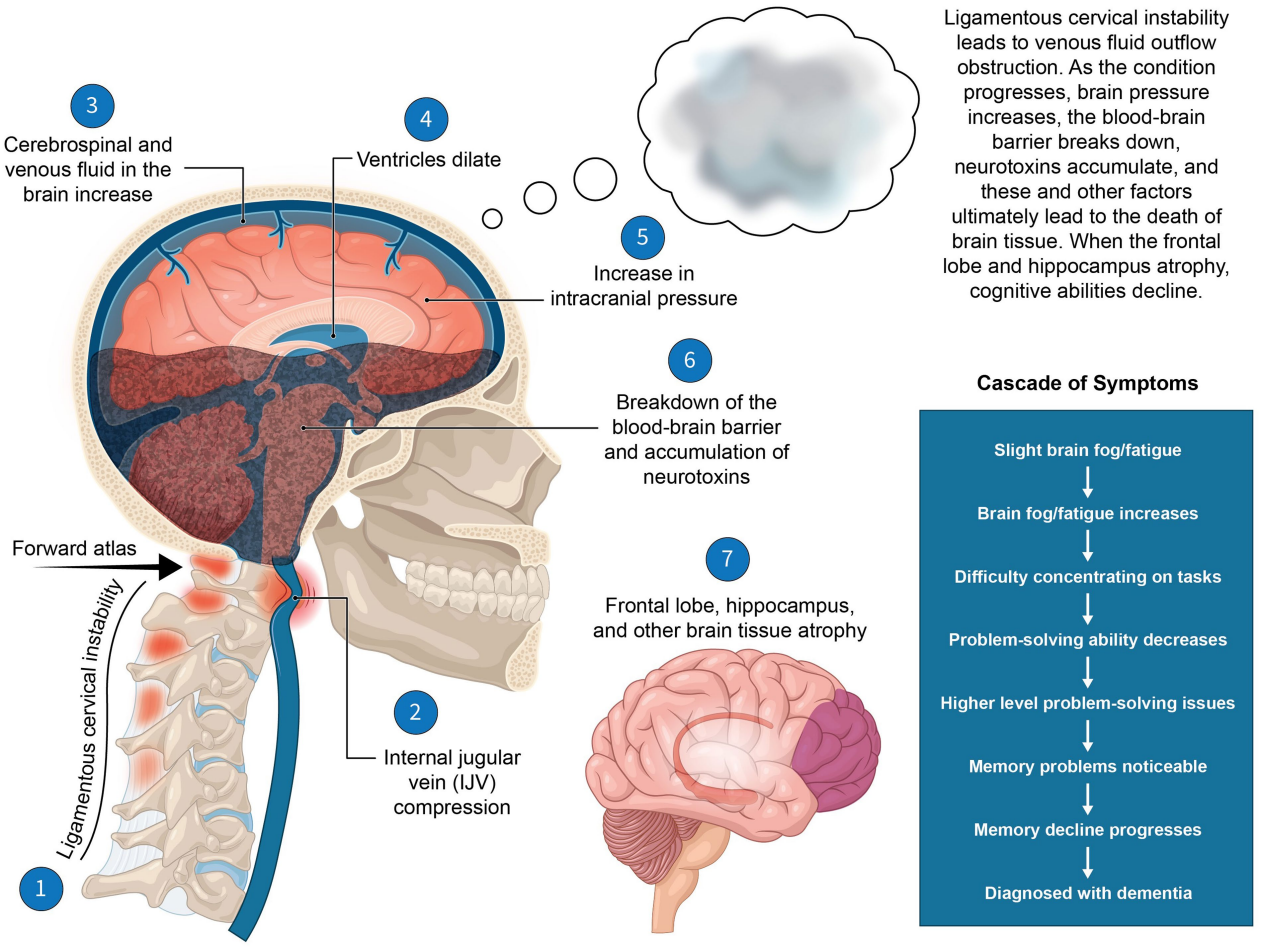
IJV compression due to unresolved ligamentous cervical instability/dysstructure can cause functional and structural brain impairment.

IJV compression was documented at the level of C1 (atlas) in 98% of this cohort of patients experiencing brain-based symptoms, while 99% displayed elevated ONSD, causing us to consider IJV compression as a potential pathophysiological explanation for otherwise elusive symptom etiology, possibly due to increased intracranial pressure (intracranial hypertension). There are many ways in which IJV compression could cause functional and structural brain impairment, including raising the brain pressure, decreasing cerebral perfusion, cerebral microvascular structure impairment, breakdown of the blood–brain barrier, and a change in cerebrospinal fluid dynamics, among others (59–61) (Figure 8).

Approximately 70–80% of intracranial fluid flows through the venous system, with around 70% of blood exiting the brain through the IJVs in the supine position (62). Compression of the IJVs in the supine position is an established cause of elevated intracranial pressure (63). Cerebral venous outflow obstructions, including those in the IJVs, are increasingly recognized in cases of intracranial hypertension that were previously identified as “idiopathic” (64, 65).

Cerebral venous outflow disorders (e.g., IJV stenosis) are known to have a variety of clinical presentations, including neurological deficits, headache, altered consciousness, tinnitus, dizziness, cognitive decline, and vision changes, as well as anxiety and depression (66, 67). Intracranial hypertension (including idiopathic) is associated with a host of brain-based symptoms ranging from psychiatric and mental health conditions to cognitive difficulty, including mental confusion, difficulty concentrating, derealization, obsessive-compulsive disorder, brain fog, irritability, anxiety, and depression (68–72). Many of these symptoms improve dramatically with restoration of IJV and cerebral venous flow (73–75). Restoration of IJV flow has also been shown to reduce brain ventricular volume by as much as 85%, improve brain perfusion, and help restore brain parenchymal volume in cases of intracranial hypertension (76–78).

The IJVs are the main outflow pathways for cerebral venous drainage in the supine position. As the cervical instability and cervical dysstructure progress, cerebrospinal fluid and blood flow into and out of the brain can become impaired, potentially resulting in poor brain function, with accompanied symptoms. If this is true, then structural cervical IJV compression should be evaluated in patients with brain-based symptoms such as brain fog, difficulty concentrating, and mental health symptoms, especially in those with neck pain and headaches.

#### 4.4.3 Optic nerve sheath diameter and elevated intracranial pressure

This patient population had a mean sum ONSD of 15.39 mm (normal sum <12.2 mm), with 99% having bilateral elevated ONSD values indicative of elevated intracranial pressure. In this study, we evaluated ONSD using a relatively high cutoff of 6.1 mm.

**Figure 9:**
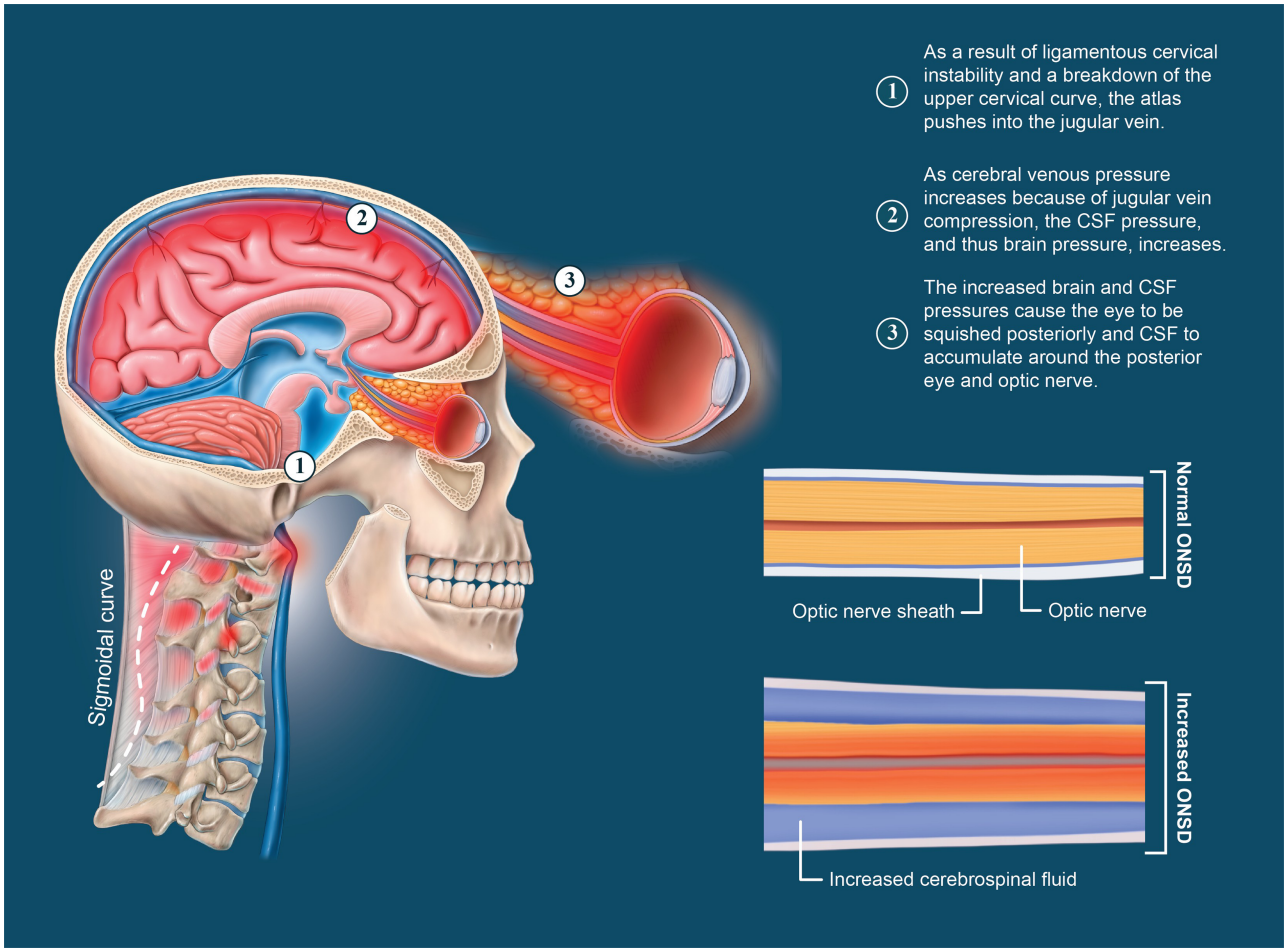
Optic nerve sheath diameter (ONSD) is indicative of elevated intracranial pressure.

Transorbital ultrasonographic examination of the ONSD is used in many settings to detect elevated intracranial pressure, as ONSD measurements are shown to correlate with elevated intracranial pressure by invasive and noninvasive measurements (79, 80). ONSD is indicative of elevated intracranial pressure because it provides a picture of increased CSF accumulated around the optic nerve through the subarachnoid space. In this patient population, elevated ONSD was not surprising given the frequency of IJV compression, which we understand is a mechanism of inhibiting cerebral venous outflow, including CSF drainage (Figure 9).

We used in-office B-mode ultrasound for real-time, noninvasive, high-resolution imaging. ONSD should be measured 3 mm behind the posterior rim of the globe, identifying the nerve sheath (hypoechoic linear structure with a hyperechoic border) that emerges from the posterior part of the globe and includes the outer rim.

The established normal ONSD value in healthy adults ranges from <5.0–5.4 mm (81). When evaluating for elevated intracranial pressure, the widely accepted cutoff value used in emergency rooms and other settings ranges from >5.6–6.0 mm, although some have found as low as >5.3–5.7 mm to correlate with elevated intracranial pressure >20 mmHg (normal is 10–15 mmHg) (82–85).

It has already been established that eye-neck integrated ultrasound of the IJVs and ONSD can help identify the IJVs as the structural mechanism causing increased intracranial pressure, differentiating it from *idiopathic* intracranial hypertension, which arises spontaneously (86). The significance of our findings is that cervical structural abnormalities could contribute to IJV compression, which could cause elevated ONSD, indicating elevated intracranial pressure. Elevated intracranial pressure caused by IJV (cerebral venous outflow) obstruction could contribute to some brain-based symptoms.

### 4.5 Vagus nerve evaluation

Vagus nerve cross-sectional areas can be easily measured by ultrasound of the carotid sheath in the cervical spine. Canon Aplio a550 ultrasound with a 7 MHz linear probe was used in this study. In patients with unidentified etiologies for various brain-based or systemic symptoms, ultrasound examinations of the vagus nerve should be included as part of a full diagnostic workup to consider sympathetic/parasympathetic imbalance as a contributing pathology (Figure 7).

Normal cross-sectional areas of the vagus nerves in healthy adults average between 1.9–2.5 mm^2^, the right often larger than the left (17, 87). This patient population presented with significantly decreased vagus nerve CSA, with averages of 1.33 mm^2^ on the right and 1.37 mm^2^ on the left. In our clinic, cervical spine-related pathology typically presents symmetrically, which would make sense if the cause of it is from a facedown/forward head lifestyle, while traumatic cervical spine injuries would more likely give asymmetric pathology. In this study, we therefore analyzed data such as the vagus nerve CSA as the bilateral sum, using a relatively low cutoff of >4.2 mm^2^ to identify abnormal. We found that 95% of patients had decreased total vagus nerve CSA, indicative of vagus nerve atrophy (degeneration). As the vagus nerves and IJVs traverse the neck just anterior to the cervical vertebrae, they, like any nerve, are subjected to injury from stretch and compression (88). Cervicovagopathy, or vagus nerve degeneration, from cervical pathology may be a contributing factor to some brain-based symptoms in this patient group (89).

#### 4.5.1 Vagus nerve and sympathetic dominance

The vagus nerves are the dominant nerves of the parasympathetic nervous system, and the primary inhibitors of sympathetic stimulation. Around 80–90% of parasympathetic nerve fibers are found in the vagus nerve (90). Degeneration or dysfunction of the vagus nerves results in reduced vagal activity, one cause of a sympathetic-dominant state. Hyperactivity of the sympathetic system and/or parasympathetic hypoactivity causes sympathovagal imbalance, something seen in a host of brain and body diseases, including mental health disorders (90–92).

Autonomic dysfunction is a common finding in people with mental health challenges such as rumination, anxiety, or depression (93–95). Autonomic nervous system dysfunction affects all the organs and systems of the body with its myriad potential origins (96).

Vagus nerve degeneration is being increasingly studied in association with brain diseases such as Parkinson’s and Alzheimer’s, as well as depression and anxiety, as decreased vagus nerve CSA in neurodegenerative patient populations correlates with parasympathetic dysfunction and emphasizes the vagus nerves’ role in autonomic modulation (97, 98). It has been indicated that ultrasound examination of vagus nerve cross-sectional area could be considered alongside more “standard” tests of autonomic nervous system function, such as the tilt test, to aid in clinical diagnosis of parasympathetic dysfunction (21).

Furthermore, vagus nerve stimulation (VNS) has been suggested as a treatment option for many brain conditions, including anxiety, depression, primary headache disorders, and cognitive disorders (99–102) While the precise mechanisms of VNS and its benefits in brain diseases are not yet fully understood, human and/or animal studies are revealing that VNS can inhibit inflammation (including brain neuroinflammation), promote neuroprotection, and help maintain the blood–brain barrier, as well as transmit signals from the gut to the brain (103, 104). It appears that VNS enhances sympathetic/parasympathetic balance by improving parasympathetic signaling. Transcutaneous or auricular vagus nerve stimulators are being used to improve alertness, problem solving, mood, and memory enhancement in such conditions as bipolar disorder, anxiety, post-stroke, Alzheimer’s disease, and other types of cognitive decline (105–107). We propose that conditions that are helped by VNS might then be caused by vagus nerve degeneration.

#### 4.5.2 Effects of autonomic nervous system imbalance

While the vagus nerves are known to be vitally important for the maintenance of body homeostasis and health, the same could be said for their role in the brain. While the exact mechanism by which VNS aids in conditions such as depression is not entirely elucidated, neuroscience (including functional neuroimaging) suggests that it aids in blood flow regulation, cerebral blood vessel diameter and tone, release of neurotransmitters, and improvement of functional brain connectivity, and stimulates various parts of the brain that improve mood (103, 108–113).

Vagus nerve degeneration can result in a cascade of outcomes due to unopposed sympathetic activity. The chronic stress state of sympathetic overdrive disrupts the hypothalamic–pituitary–adrenal axis, causing headaches due to neuroinflammation and vascular changes, and can create a cycle of added stress from the heightened pain sensitivity and increased frequency and severity of headaches (114).

As vagus nerve research evolves, we highly suggest more rigorous studies to not only evaluate vagus nerve function, but also size, as ultrasound measurements of vagus nerve CSAs are noninvasive and easy to perform. When small vagus nerve CSAs are found in the context of LCI and cervical dysstructure, then cervical curve correction could lead to improvement of vagus nerve activity. We suspect that by relieving the vagus nerve of excessive stretch or compression, symptoms will improve or resolve.

### 4.6 Pupillary insights into autonomic balance

Pupillometry has been used to document such conditions as acute rises in intracranial pressure, as occurs after trauma or in intensive care units after brain surgery; it is also a nonspecific indicator of sympathetic hyperactivity (115, 116). In this patient population with brain-based symptoms, 95% exhibited the high end of pupillary light constriction percentage, with a mean of bilateral total of 74.4%, (normal 30–60%), and 91% had relatively large pupil diameters, with the cohort’s average bilateral pupil diameter being 10.22 mm (normal bilateral sum is 4–8 mm). While pupillary light constriction percentage typically falls between 15 and 30% (unilateral), a bilateral total exceeding 70% may not always be abnormal. Given that the baseline pupil diameters are also at the high end of the normal range before stimulus, however, we consider these results to be indicative of intact pupillary response, but possibly in line with baseline sympathetic-dominant states (117).

### 4.7 Tonometry

Tonometry, which measures intraocular pressure, is also part of the neck vitals analysis, as elevation of intracranial pressure has many effects on the eye, including posterior globe flattening, modulation of aqueous humor dynamics, and elevations of eye pressure (118–120). It is easy to document the relationship between the jugular veins and intraocular pressure, as simply wearing a necktie can increase pressure by 2–4 mmHg (121–123). In about two-thirds of cases, a rise in intraocular pressure is associated with a rise in intracranial pressure (124). It should also be remembered that the aqueous outflow of the eye is through the episcleral veins, which drain into the cavernous sinus before flowing into the IJVs. In our clinical cases with elevated intraocular pressure (and the patients had no intrinsic eye pathology) where IJV compression was resolved by neck reconstruction therapy, the elevated intraocular pressures diminished. This patient population tended to have higher-than-normal eye pressures 17.8 mmHg per eye, or 35.6 mmHg total for both eyes (normal intraocular pressure is 10–15 mmHg), with 19% having bilateral total eye pressures greater than 42 mmHg (>42 mmHg indicates ocular hypertension).

### 4.8 Theoretical considerations

The concept of “brain-based symptoms” is nonspecific and can arise from a variety of etiologies and contributing factors, one of which could be cervical structural pathology (1). It is emphasized that the findings in this study do not establish that LCI causes internal jugular venous obstruction, intracranial pressure elevation, or brain dysfunctions or symptoms, but rather, the findings suggest possible associations in this particular patient population. Given the nature of the human body and the cervical spine in particular, the multifaceted hypothesis presented in this paper acknowledges that ligamentous cervical instability and cervical dysstructure could affect brain health and a person’s response to stress via multiple mechanisms, including IJV compression and vagus nerve degeneration/dysfunction. If these conditions are in fact caused by compressive pressure from abnormal cervical bony anatomy, then the severity of symptoms, and even which symptoms present each day, can vary with the change in bony anatomy. Improvement in various types of cervical structural dysfunctions, including forward head posture, loss of cervical lordosis, and instability by such modalities as physiotherapy, Prolotherapy, and surgical stabilization have resulted in significant improvements in neck pain, anxiety, depression, and overall disability (41, 125–128).

**Figure 10:**
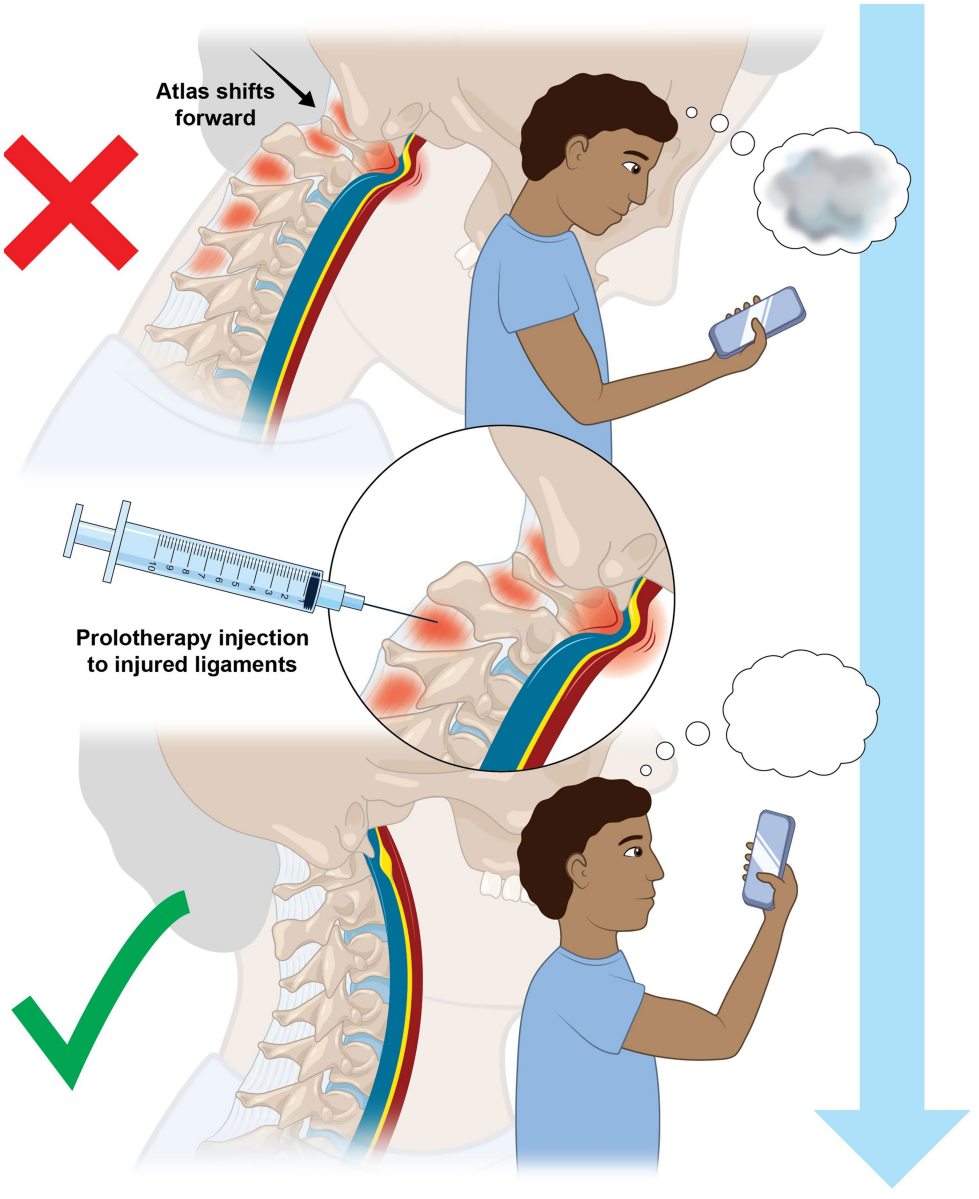
Curve correction, lifestyle modifications (such as looking up while using screens), and various therapies, including prolotherapy to strengthen the ligaments, are part of a dynamic structural medicine protocol aimed at relieving neurovascular compressions to resolve chronic brain symptoms.

A dynamic structural medicine protocol for brain symptoms due to cervical spine abnormalities could include recommendations such as postural ergonomic changes when using electronic devices such as computers and cell phones, physical therapy, gentle chiropractic or osteopathic adjustments, therapeutic exercises, use of a cervical lordotic device such as the Denneroll^®^, and regenerative therapies like Prolotherapy to assist in cervical curve stabilization by strengthening the ligaments (Figure 10).

### 4.9 Limitations

One limitation of this study is the nature of a retrospective design, making it difficult to control for potential confounders such as lifestyle (posture, work environment, etc.). All patients in this study reported to an outpatient neck clinic with no known etiology after seeing multiple specialists, therefore the results may not be applicable to patients with other known origins of their symptoms or overlapping conditions. This patient group may not represent the general population or account for *all* causes of the symptoms being considered here. This cohort may, however, represent a significant group of patients who experience brain-based symptoms without a known cause. Since there were no “asymptomatic” or “normal cervical lordotic curve” control groups for comparison, the ability to differentiate findings was limited. The study did not include follow-up data to evaluate symptom progression or response to treatment. The extent of uniformity and diagnostic workups prior to reporting to this clinic may vary, as *we relied on patient reporting to identify no previously known cause.* The study did not aim to evaluate mechanistic insights into individual symptoms or establish causality, but instead aimed to identify patterns across the entire cohort to encourage further research. The exact amount of time or posture of the participants while using computers or cell phones was not documented. The facedown/forward head lifestyle as a contributing factor is speculative, and further research should consider documenting detailed lifestyle factors contributing to symptom development.

## 5 Conclusion

In this cohort of 227 patients presenting with brain-based symptoms at an outpatient neck clinic, radiologically identified forward head posture, loss of lordosis, and ligamentous cervical instability were nearly ubiquitous. Concurrently, IJV compression, vagus nerve degeneration, and elevated ONSD were observed in almost all patients. This cross-sectional study identifies clinical associations between presenting symptoms and initial test findings, suggesting that cervical pathology may be an etiological basis of, or a contributing factor to, not only musculoskeletal symptoms such as neck pain and headache, but also mental health conditions, including anxiety, depression, brain fog, difficulty concentrating, obsessive thoughts, and rumination. Further longitudinal studies are necessary to evaluate these findings on an individual-symptom basis.

## Data Availability

The raw data supporting the conclusions of this article will be made available by the authors, without undue reservation.
