# Supplementary figures and images for: Brain-based symptoms reported in young adults evaluated for cervical spine etiology: a retrospective, cross-sectional clinical study with preliminary data on over 200 patients
Source: Front Neurol. 2026 May 25;17:1749266. doi: 10.3389/fneur.2026.1749266 (PMC13245198; doi:10.3389/fneur.2026.1749266)

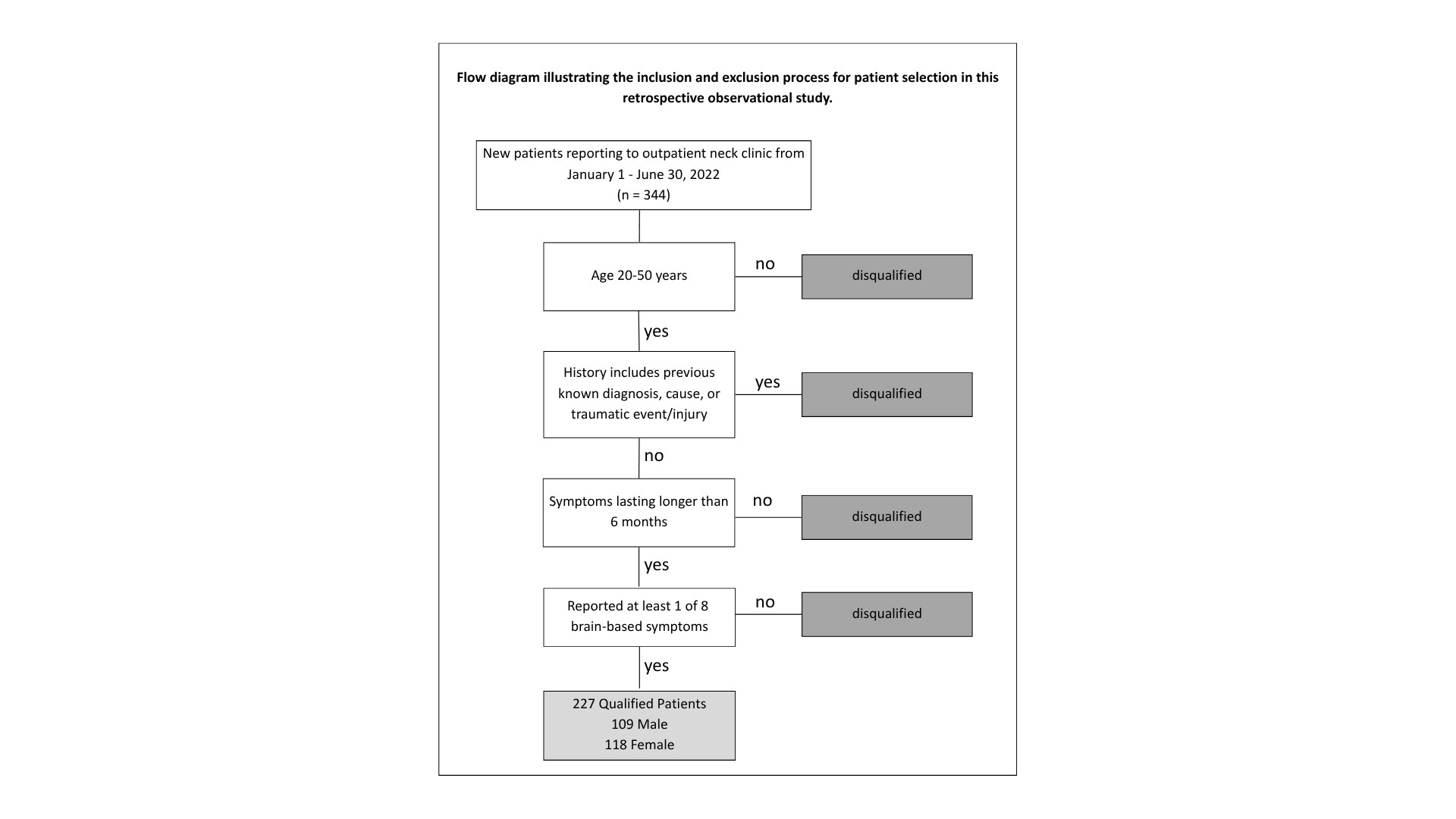

Supplement: Supplementary file 1 [file Image_1.jpeg]
